# *In Vitro* Effects of Doxycycline on Replication of Feline Coronavirus

**DOI:** 10.3390/pathogens10030312

**Published:** 2021-03-07

**Authors:** Magdalena Dunowska, Sayani Ghosh

**Affiliations:** School of Veterinary Science, Massey University, Palmerston North 4410, New Zealand; S.Ghosh1@massey.ac.nz

**Keywords:** feline infectious peritonitis (FIP), feline coronavirus, doxycycline, antiviral

## Abstract

Feline infectious peritonitis (FIP) is a sporadic fatal disease of cats caused by a virulent variant of feline coronavirus (FCoV), referred to as FIP virus (FIPV). Treatment options are limited, and most of the affected cats die or are euthanized. Anecdotally, doxycycline has been used to treat FIP-affected cats, but there are currently no data to support or discourage such treatment. The aim of this study was to establish whether doxycycline inhibits replication of FIPV *in vitro*. The virus was cultured in Crandell-Rees feline kidney cells with various concentrations of doxycycline (0 to 50 µg/mL). The level of FIPV in cultures was determined by virus titration and FCoV-specific reverse-transcription quantitative PCR. Cell viability was also monitored. There was no difference in the level of infectious virus or viral RNA between doxycycline-treated and untreated cultures at 3, 12- and 18-hours post-infection. However, at 24 h, the growth of FIPV was inhibited by approximately two logs in cultures with >10 µg/mL doxycycline. This inhibition was dose-dependent, with inhibitory concentration 50% (IC_50_) 4.1 µg/mL and IC_90_ 5.4 µg/mL. Our data suggest that doxycycline has some inhibitory effect on FIPV replication *in vitro*, which supports future clinical trials of its use for the treatment of FIP-affected cats.

## 1. Introduction

Feline infectious peritonitis (FIP) is a sporadic fatal disease of cats caused by a virulent variant of feline coronavirus (FCoV), referred to as FIP virus (FIPV) [1]. Feline coronavirus is classified within the genus *Alphacoronavirus* in the family *Coronaviridae* of the order *Nidovirales* [2]. With a few exceptions where a horizontal spread was suspected [3], FIPV is believed to emerge *de novo* via mutations of the relatively non-pathogenic variant of FCoV, designated feline enteric coronavirus, within individual hosts [4,5]. It is generally well accepted that the acquisition of macrophage tropism is an essential step in the evolution of FECV into FIPV, resulting in transition from a largely subclinical localized enterocyte infection to a systemic monocyte/macrophage infection with a fatal outcome. However, factors that determine this switch are currently poorly understood [4]. Young animals from a multi-cat environment with high FCoV antibody titres seem to be at increased risk of developing FIP [6]. Despite many attempts to produce a vaccine to help control FIP, only one is available, with limited efficacy [7]. While some cats appear to be more resistant to FIP than others under experimental conditions [4], treatment options for field cases are limited. The vast majority of the affected cats either die within weeks to months following the first appearance of clinical signs or are euthanized. Hence, FIP remains one of the leading causes of death among young cats worldwide, particularly those in shelters and catteries. 

Owners who acquire a young cat often become very attached to it before the first clinical signs of FIP develop. Hence, despite the very poor prognosis for such cats, many elect to attempt treatment. The protocols used depend on clinical presentation, which can vary considerably [8,9], but generally include supportive therapy (e.g. draining of the effusions) and the use of drugs designed to modulate the immune responses to FIPV infection. The latter involves either dampening the ineffective humoral responses that contribute to the disease process or non-specific stimulation of the cellular immune responses, which are believed to be important in protection/recovery from coronavirus infections [4,10]. The drugs that have been used for these purposes include prednisolone, polyprenyl immunostimulant (PI), feline interferon omega, cyclophosphamide, itraconazole, pentoxyfilline or propentofylline [11,12,13,14,15]. However, there is little evidence to suggest that these treatments are effective [8]. The willingness of the owners to hold on to the tiniest glimmer of hope offered by any attempt at treatment can be illustrated by the fact that the global surge in the use of PI was sparked by the description of its apparent effectiveness following treatment of only three cats with a mild, dry form of FIP [12]. Recently, a nucleoside analog (Remdesivir, GS-5734) has been authorized by the United States Food and Drug Administration for emergency use in hospitalized patients affected by coronavirus disease 2019 (COVID19) [16]. A very similar compound that served as an intermediate in the production of Remdesivir (GS-441524) has been shown to be effective at treating FIP-affected cats [17,18,19]. In addition, a viral protease inhibitor (GC376) also showed some efficacy against FIPV [20,21]. While this has raised hopes of many cat owners and FIP researchers worldwide, none of these antiviral drugs are currently commercially available for veterinary use [22]. 

Anecdotally, doxycycline has been used to treat FIP-affected cats by some veterinarians in the USA and Australia. Doxycycline belongs to the tetracycline class of antimicrobial drugs with broad spectrum of activity against Gram-positive and Gram-negative bacteria. The mechanism of antibacterial action of tetracyclines is mainly related to their ability to inhibit protein synthesis through binding to the bacterial 30S ribosomal subunit [23]. However, tetracyclines have also been shown to have other beneficial biological properties, not directly linked to their antimicrobial use. These include anti-inflammatory, anti-apoptotic, antiparasitic and antiviral activity [24,25,26].

Although there are currently no scientific data to support the use of doxycycline for treatment of FIP, tetracyclines (doxycycline and minocycline) have been shown to be effective in inhibiting replication of several other viruses both *in vitro* [27,28,29,30,31,32,33] and *in vivo* [34,35,36,37]. The exact mechanism of the antiviral properties of doxycycline are not well understood, but they may be related to its ability to inhibit matrix metalloproteinases [38,39,40,41] or to bind double stranded RNA [42].

Re-purposing of an already available drug such as doxycycline for the potential treatment of FIP would have several advantages: doxycycline is already easily available, comparatively cheap, and its safety for use in cats is well established. However, there are currently no scientific data available to support (or discourage) such use. The work presented in this paper addresses this knowledge gap. The aim of the study was to establish whether or not doxycycline inhibits *in vitro* replication of FIPV. 

## 2. Results

### 2.1. Doxycycline Toxicity

Doxycycline was not toxic to the Crandell-Rees feline kidney (CRFK) cells at concentrations ranging from 5 to 50 µg/mL, and hence all tested concentrations were used in the antiviral activity testing.

### 2.2. Levels of Infectious FIPV and Viral RNA in Cultures with and without Doxycycline

**Figure 1:**
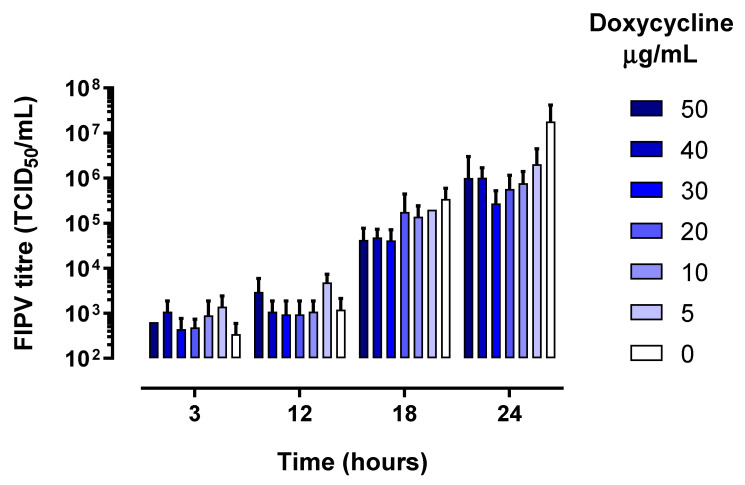
Feline infectious peritonitis virus (FIPV) titre in cell culture lysates collected at various time points stratified by concentration of doxycycline in the growth media. The error bars represent standard deviation.

**Figure 2:**
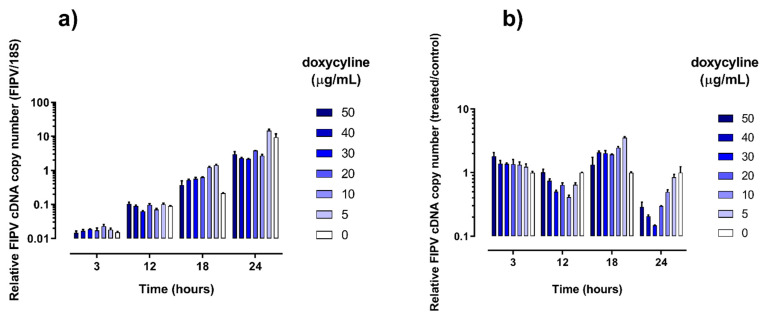
Copy numbers of feline infectious peritonitis virus (FIPV) RNA in cell culture lysates collected at various time points stratified by concentration of doxycycline in the growth media. Results are expressed as calculated concentration of FIPV cDNA divided by the mean calculated concentration of 18S ribosomal cDNA in the same wells (**a**) or as calculated concentration of FIPV cDNA grown in the presence of doxycycline (treated) divided by the calculated concentration of FIPV cDNA grown without addition of doxycycline (control) (**b**). All quantitative PCR tests were run in duplicate. The error bars represent standard deviation.

As expected, the levels of infectious virus increased in all wells between 3 and 24 h of culture (Figure 1), which was accompanied by a corresponding increase in the levels of FIPV RNA (Figure 2). 

**Figure 3:**
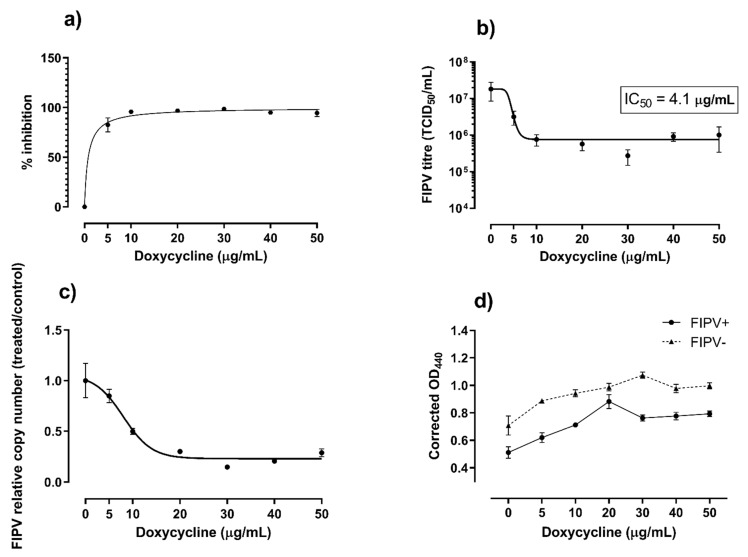
Summary of data from the 24-hour cultures. Percent inhibition (**a**) and reduction in titre (**b**) of feline infectious peritonitis virus (FIPV) cultured in the presence of various concentration of doxycycline, as indicated. The corresponding relative copy number of viral RNA (**c**) and viability of Crandell-Rees feline kidney cells (**d**) are also shown. The cell viability was determined using WST-1 reagent and expressed as corrected optical density (OD) values at 440 nm. The corrected OD_40_ values are proportional to the numbers of viable cells. Error bars represent standard error of the mean.

There was no difference in the levels of infectious virus or viral RNA between doxycycline-treated and untreated samples at 3-, 12- and 18-hours post-infection. However, at 24 h, the growth of FIPV was inhibited 94% to 98% in the presence of doxycycline at ≥10 µg/mL (Figure 3a). The corresponding FIPV titres were about 2 logs lower than the titre of the virus in the untreated wells (*p* < 0.001, Figure 3b) and the levels of viral RNA were significantly (*p* < 0.05) lower in wells with all concentrations of doxycycline tested compared to untreated wells (Figure 3c). The inhibition of FIPV growth in CRFK cells at 24 h post-infection was dose-dependent, with median inhibitory concentration (IC_50_) of 4.1 µg/mL (8.0 µM) and 90% inhibitory concentration (IC_90_) of 5.4 µg/mL (10.5 µM).

### 2.3. Viability of CRFK Cells Infected with FIPV in Cultures with and without Doxycycline

**Figure 4:**
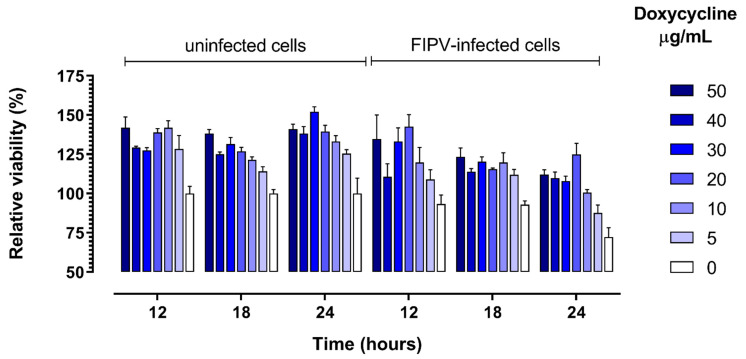
Relative viability of cells cultured in the presence of 0 to 50 µg/mL of doxycycline and control uninfected cells at specified times post-infection with feline infectious peritonitis virus (FIPV). The viability was determined using WST-1 reagent and expressed as percent of viable cells relative to the number of viable cells in uninfected cultures without addition of doxycyline at each timepoint. Error bars represent standard error of the mean.

As expected, the viability of CRFK cells in wells that were inoculated with FIPV decreased over time as compared with control uninfected cells. The viability of cells was higher in the presence of 10 to 50 µg/mL doxycycline in comparison with control cells cultured without addition of doxycycline for both virus-infected and non-infected cells at all three time points (12 h, 18 h and 24 h) (Figure 4). This positive effect of doxycycline on cell viability was the strongest at 24 h post-infection, when it was apparent for all (5 to 50 µg/mL) doxycycline concentrations tested (p = 0.036, Figure 3d).

## 3. Discussion

We have shown that addition of doxycycline to cell culture has an inhibitory effect on replication of FIPV. The highest difference in the virus titre between treated and untreated cultures was about 2 logs, with a similar fold difference in the levels of viral RNA. While it is difficult to directly compare these results with antiviral effects of doxycycline reported by others, similar levels of inhibition of *in vitro* viral growth were reported for porcine reproductive and respiratory syndrome virus (PRRSV) grown in MARC-145 cells for 36 h [27] or vesicular stomatitis virus (VSV) grown in Vero cells for 24 h [31]. 

This level of inhibition of the *in vitro* growth of PRRSV and VSV was apparent in the presence of only 1 µg/mL doxycycline, in contrast to the inhibitory effect against FIPV virus, which was most noticeable at concentrations at or above 10 µg/mL. The IC_50_ calculated in the current study (4.1 µg/mL, 8.0 µM) was higher than IC_50_ reported for PRRSV (0.25 µg/mL) [27], VSV (0.18 µg/mL) [31], or severe acute respiratory syndrome CoV-2 (SARS-CoV-2) (4.5 µM) [33]. The average plasma concentration of doxycycline administered to cats intravenously at dosages between 2.5 and 10 mg/mL every 12 to 24 h was reported to be between 1.59 and 6.37 µg/mL, depending on the dosage regimen [43]. Such concentrations may be too low to have an inhibitory effect based on results of the current study. However, the corresponding maximum plasma concentrations (Cmax) varied from 20.90 to 83.76 µg/mL, with calculated Cmax/IC_50_ values between 5.1 and 20.4 and Cmax/IC_90_ values between 3.9 and 15.5, which may be high enough to induce potential inhibitory effect *in-vivo*. However, doxycycline reached the maximum concentration of only about 4 µg/mL (Cmax/IC_50_ of 0.97) after oral administration of 5 mg/mL [44]. Altogether, the currently available data suggest that it may be difficult to achieve plasma concentrations of doxycycline that would have a potential inhibitory effect on FIPV replication for a prolonged period of time, particularly after oral administration. However, since the *in vivo* interactions between the virus and the host are more complex than those observed *in vitro* [45], the effects of doxycycline on FIPV replication and disease progression *in-vivo* need to be further evaluated in clinical trials. Such trials should involve cats infected with both type I and type II FCoV. Most *in vitro* research, including the current study, was done with type II FCoV because, unlike type I FCoV, it grows well in cell culture. The majority of field FIP cases are, however, associated with infection with type I FCoV [46], which may differ in its response to doxycycline treatment.

The exact mechanism of antiviral effects of doxycycline is not fully understood and appears to differ between viruses. Doxycycline was shown to have inhibitory effects at both entry and post-entry stages of *in vitro* replication of several viruses, with stronger effects exhibited at the adsorption/entry stage than at the replication stage for Chikungunya virus [34], Dengue virus [29], and SARS-CoV-2 [33]. In contrast, its inhibitory action was linked mainly to early replication stages of VSV [31] and PRRSV [27], with no effect on virus entry. These discrepancies may represent true differences between doxycycline interactions with different viruses, but they may also reflect differences in the experimental design used. In the current study doxycycline was added to the cultures at the time of virus infection, and hence we were not able to differentiate between entry and post-entry effects. Interestingly, results of *in silico* analyses suggested that doxycycline may efficiently bind spike protein [47] and main proteinase [48] of SARS-CoV-2. While the implications of these findings for the *in vivo* use of doxycycline against SARS-CoV-2 and other coronaviruses remain to be established, such interactions, if true, are compatible with inhibition of the virus replication at both entry (spike protein binding) and early-replication (main proteinase binding) stages of infection.

Doxycycline appeared to have a positive effect on CRFK cell viability *in vitro* within the concentrations tested. This was in contrast to reports of inhibition of mitochondrial protein synthesis accompanied by reduced oxygen and increased glucose consumption as well as increased lactate production rates in several human cell lines cultured in the presence of 1 µg/mL doxycycline [49,50]. Doxycycline seemed to impair proliferation of human cell lines at concentrations up to 10 µg/mL and showed toxic effects at 10 µg/mL. Others reported that 50% cytotoxic concentration of doxycycline in Vero E7 cells were >100 µg/mL [33]. In addition, doxycycline at 0.1–1 µg/mL, but not at 10 µg/mL, protected human glioma cells from hypoxia-induced cell death, although none of the concentrations tested (0.1–10 µg/mL) had any effect on cell viability under normoxic conditions [50]. Supplementation with 1 µg/mL doxycycline had strong positive effect on survival and self-renewal of embryonic stem and induced pluripotent stem cells through activation of a PI3K-AKT intracellular signaling [51]. Altogether, these data suggest that while doxycycline affects cell metabolisms *in vitro*, the exact effects of such interactions may vary between different cell lines and under different experimental conditions. The possible influence of such interactions on results of *in vitro* studies should be kept in mind. Hence, we cannot exclude that some of the apparent antiviral effects observed in the current study reflected the effects of doxycycline on survival/metabolism of CRFK cells, which may have indirectly affected replication of FIPV. 

It has been suggested that doxycycline may have beneficial effects for people affected by the newly emerged coronavirus disease 2019 (COVID19) due to its antiviral and anti-inflammatory properties [52,53]. The latter seem particularly relevant, as dysregulation of the innate immune responses and the cytokine storm have been associated with severe COVID19 cases [54]. This notion may be supported by observational reports of apparent benefits of the use of doxycycline for treatment of COVID19 patients [55], despite the fact that the *in vitro* calculated ratios of Cmax/IC_50_ and Cmax/IC_90_ seemed too low to reach effective concentrations in human plasma to inhibit growth of SARS-CoV-2 in [33]. As the deregulation of the immune responses is a common feature in coronavirus-induced diseases including FIP [56,57], the anti-inflammatory effects of doxycycline (not investigated in the current study) may be relevant for treatment of FIP-affected cats.

In conclusion, our results extend those recently reported by Gendrot and others [33] on the inhibitory effects of doxycycline on the *in vitro* replication of SARS-CoV-2 into another coronavirus from a different genus (alphacoronavirus versus betacoronavirus) and provide experimental support for further *in vivo* trials to establish whether or not doxycycline could be of benefit for treatment of FIP-affected cats. 

## 4. Materials and Methods

### 4.1. Doxycycline Toxicity 

Toxicity of doxycycline for cultured cells was established using cell proliferation reagent WST-1 (Roche, Rotkreuz, Switzerland, according to the manufacturer’s instructions. Doxycycline (doxycycline hyclate, Sigma-Aldrich, St. Louis, MO, USA) was diluted to a concentration of 50 mg/mL in water and this preparation was added to the growth media (GM) to the final desired concentration (5 to 50 µg/mL). This range of doxycycline concentrations was chosen as it encompasses concentrations that are achievable in sera following administration of the recommended dose (2.5 to 10 mg/kg) to cats [43]. 

### 4.2. Production of the Virus Stock

A tissue culture adapted strain of type II FIPV (sourced from American Tissue Culture Collection, Manassas, VA, USA) was propagated and titrated in CRFK cells. Cells were maintained in the GM, which comprised Advanced Dulbecco’s modified Eagle’s medium (DMEM, ThermoFisher Scientific, Waltham, MA, USA) supplemented with 2% foetal calf serum (ThermoFisher Scientific), 1% antibiotic solution (PenStrep, ThermoFisher Scientific) containing 10,000 units/mL of penicillin and 10,000 µg/mL of streptomycin, and 1% Glutamax (ThermoFisher Scientific). 

### 4.3. Design of the Study

CRFK cells were seeded into four replicate 24-well plates at a seeding density of 10^5^ cells/0.5 mL per well. The GM was the same as that used for the production of the virus stock, with the exception that PenStrep was replaced with various concentrations of doxycycline (50, 40, 30, 20, 10, and 5 µg/mL). The wells were inoculated with 10^4^ tissue culture effective dose 50% (TCID_50_) of FIPV (multiplicity of infection (moi) 0.1) in triplicate at the time of seeding and incubated at 37 °C in 5% CO_2_ for 24 h. Cell controls (non-inoculated cells maintained in GM without doxycycline) and virus controls (FIPV-inoculated cells maintained in GM without doxycycline) were included on each plate. One plate was frozen at −80 °C at each of the predetermined time points (3, 6, 18 and 24 h). The plates were then thawed and the content of each well aliquoted and re-frozen until it was used for virus titration and FCoV-specific reverse-transcription quantitative PCR (RT-qPCR). 

### 4.4. Virus Titration

The virus was titrated using standard virological protocols. Briefly, serial 10-fold dilutions of the virus in GM (50 µL/well) were made in 96-well tissue culture plates in duplicate (Nunc, ThermoFisher Scientific) and CRFK cells (1:5 split) were then added to each well. Toxicity control (cells maintained in GM without the addition of the virus) and virus control (FIPV of a known titre) were included with each titration. The plates were assessed for the presence of viral cytopathic effect after 3 days of incubation at 37 °C in 5% CO_2_. The titres were calculated using the method of Reed and Muench [58]. Replicate wells showing more than 1 log difference in titre were re-titrated. The repeated titrations of the same well were removed from the analyses if they were >1 log difference from each other, so that the final average titres were calculated based on a total of 3 to 9 replicates. Percent inhibition was calculated as % inhibition = (1−(treated/control)) × 100, where “treated” represents a titre of the virus cultured in the presence of doxycycline and “control” represents an average titre of the virus grown without addition of doxycycline at the same timepoint.

### 4.5. RT-qPCR

Aliquots cell lysates from all three replicates for each time point/doxycycline concentration were pooled for RNA extractions using NucleoMag Vet (Macherey-Nagel, Düren, Germany) and KingFisher DueFlex instrument (ThermoFisher Scientific) according to the manufacturer’s instructions. Complementary DNA was prepared using a commercially available master mix (qScript cDNA SuperMix, Quanta Biosciences, Gaithersburg, MD, USA) using 8 µL RNA in a 10 µL volume according to the manufacturer’s instructions. The presence of amplifiable nucleic acids in cDNA samples was assessed by PCR with primers targeting 18S ribosomal RNA (18S rRNA) transcript [59]. All real-time reactions were performed using a commercial master mix (PowerUp SYBR green master mix, ThermoFisher Scientific), relevant primers (final concentrations 0.8 µM for FIPV primers or 0.4 µM for 18S rRNA primers, Table 1), and 2 µL of template in the total volume of 10 µL, with the following cycling profile: Uracyl-DNA glycosylase (UDG) activation at 50 °C for 2 min, polymerase activation at 95 °C for 2 min, 40 cycles of denaturation at 95 °C for 2 s and annealing/extension with data acquisition at 60 °C for 30 s, followed by a melt analysis at 3 °C/s. The FCoV primers targeting the membrane glycoprotein were designed in Geneious software version 9.1.8 (available from www.geneious.com) (accessed on 6 March 2021) and the qPCR was optimized using standard curves prepared using pre-defined concentrations (1 to 10^7^ copies/µL of template) of a target DNA (linearized recombinant plasmid that contained the target sequence).

**Figure 5:**
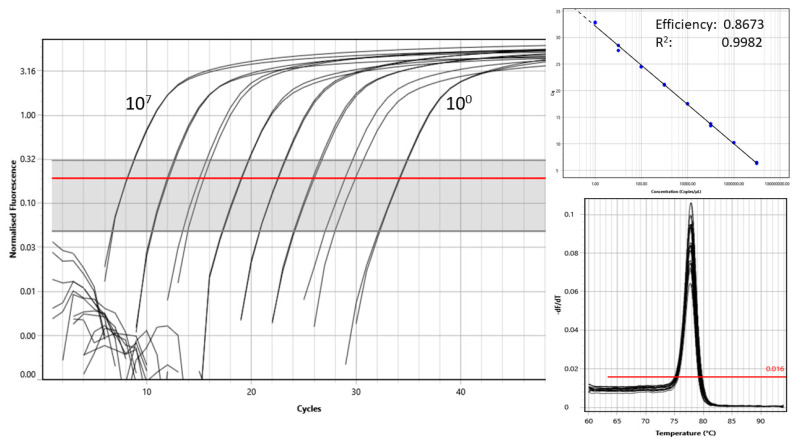
An amplification plots (**left**) and a corresponding standard curve (**top right**) and melting curves (**bottom right**) generated using standards (recombinant plasmid) containing between 10^7^ and 10^0^ copies of the target feline coronavirus sequence. The threshold (red line) was set using default settings.

The sensitivity and specificity of the assay were assessed based on the limit of detection of the FIPV standards and BLAST searches, respectively. An example of a typical standard curve is shown in Figure 5. The samples were considered positive if the amplification plots rose above the threshold, with a corresponding melting peak within 1 °C of the peak produced by the positive control. The copy number of FIPV or 18S rRNA (house-keeping gene) were calculated based on standard curves prepared for each target using plasmid DNA for FIPV and cDNA from CRFK cells for 18S rRNA. The relative quantity of FIPV cDNA was expressed as FIPV calculated copy numbers in cultures with a specific concentration of doxycycline divided by FIPV calculated copy numbers in control cultures without addition of doxycycline at the same time point. 

### 4.6. Cell Viability

In addition to 24-well plates, three 96-well plates were set-up for cell viability testing. Wells were seeded with 2 × 10^4^ cells/well (100 µL) and inoculated with FIPV at 0.1 moi at the time of seeding. For each concentration of doxycycline (0 to 50 µg/mL), 4 wells were inoculated with the virus and 4 wells were retained as non-inoculated controls. Media only wells containing GM with various concentration of doxycycline in duplicate were also set up to serve as background controls. At predetermined time points (12 h, 18 h and 24 h), 100 µL of WST-1 reagent (Roche was added to all wells on one plate and that plate was incubated at 37 °C in 5% CO_2_ atmosphere for 4 h, at which time the optical density (OD) was measured using 440 and 660 nm filters. The corrected OD_440_ values were calculated by first subtracting OD_660_ from OD_440_ value for each well (OD_440–660_), averaging the OD_440–660_ values for replicate wells, and finally subtracting the average OD_440–660_ of media only wells from the average OD_440–660_ of test wells with the same concentration of doxycycline.

### 4.7. Statistical Analysis

The measured values are presented as mean ± standard deviations or standard error of the mean, as indicated. Fisher’s exact test and one- or two-way analysis of variance (ANOVA) were used for significance analysis. A value of *p* < 0.05 was considered statistically significant. All statistical analysis including calculation of IC_50_ and IC_90_ were done using GraphPad Prism (version 9.0 for Windows, GraphPad Software, San Diego, CA, USA, available from www.graphpad.com) (accessed on 6 March 2021).

## Figures and Tables

**Table 1:** Primers used for quantitative PCR for detection of feline infectious coronavirus (FIPV) and house-keeping transcript of 18S ribosomal RNA (18S rRNA) that were used in the study.

Assay Target	Primers	Specific Product	
		Size (Base Pairs)	Tm
18S rRNA	18SrRNA.F: GTA ACC CGT TGA ACC CCA TT 18SrRNA.R: CCA TCC AAT CGG TAG TAG CG	151	83.3 °C
FIPV	26,957F_FCoV: GCA CGT ACT GAC AAT TTG AGT GAA CA 27,054R_FCoV: TCT CCC CAG TTG ACG CGT TGT	98	77.7 °C

## Data Availability

Data are contained within the article.
